# Supplementary material for: Multi‐dataset identification of innovative feature genes and molecular mechanisms in keratoconus
Source: J Cell Mol Med. 2024 Sep 19;28(18):e70079. doi: 10.1111/jcmm.70079 (PMC11412914; doi:10.1111/jcmm.70079)
Supplement: Supplementary file 2 — Material S2. [file JCMM-28-e70079-s002.zip › 7.GSEA analysis/4.MYRF/KEGG_CYSTEINE_AND_METHIONINE_METABOLISM.html]

Details for gene set KEGG\_CYSTEINE\_AND\_METHIONINE\_METABOLISM[GSEA]

|  || Dataset | MYRF.group.cls#h\_versus\_l |
| Phenotype | group.cls#h\_versus\_l |
| Upregulated in class | h |
| GeneSet | KEGG\_CYSTEINE\_AND\_METHIONINE\_METABOLISM |
| Enrichment Score (ES) | 0.24591129 |
| Normalized Enrichment Score (NES) | 1.099596 |
| Nominal p-value | 0.32179227 |
| FDR q-value | 1.0 |
| FWER p-Value | 0.998 |
Table: GSEA Results Summary

  

Fig 1: Enrichment plot: KEGG\_CYSTEINE\_AND\_METHIONINE\_METABOLISM      
 Profile of the Running ES Score & Positions of GeneSet Members on the Rank Ordered List

  

| SYMBOL | TITLE | RANK IN GENE LIST | RANK METRIC SCORE | RUNNING ES | CORE ENRICHMENT || 1 | TAT | na | 1333 | 0.416 | 0.0724 | Yes |
| 2 | BHMT | na | 2233 | 0.339 | 0.1365 | Yes |
| 3 | IL4I1 | na | 4670 | 0.233 | 0.1282 | Yes |
| 4 | LDHAL6A | na | 5245 | 0.215 | 0.1689 | Yes |
| 5 | MTR | na | 6314 | 0.183 | 0.1867 | Yes |
| 6 | MTAP | na | 6993 | 0.164 | 0.2109 | Yes |
| 7 | CDO1 | na | 7233 | 0.158 | 0.2459 | Yes |
| 8 | LDHC | na | 11201 | 0.075 | 0.1515 | No |
| 9 | GOT2 | na | 11639 | 0.069 | 0.1572 | No |
| 10 | MPST | na | 11679 | 0.068 | 0.1741 | No |
| 11 | DNMT3A | na | 12610 | 0.055 | 0.1620 | No |
| 12 | MAT1A | na | 15092 | 0.027 | 0.0976 | No |
| 13 | GOT1 | na | 16062 | 0.017 | 0.0743 | No |
| 14 | DNMT3L | na | 17060 | 0.008 | 0.0477 | No |
| 15 | MAT2A | na | 17282 | 0.006 | 0.0429 | No |
| 16 | AHCY | na | 17388 | 0.005 | 0.0413 | No |
| 17 | AHCYL1 | na | 18838 | -0.009 | 0.0018 | No |
| 18 | TRDMT1 | na | 20132 | -0.024 | -0.0292 | No |
| 19 | MAT2B | na | 21416 | -0.038 | -0.0559 | No |
| 20 | ADI1 | na | 21902 | -0.044 | -0.0581 | No |
| 21 | AMD1 | na | 22622 | -0.054 | -0.0645 | No |
| 22 | SMS | na | 22809 | -0.057 | -0.0547 | No |
| 23 | LDHB | na | 23110 | -0.061 | -0.0471 | No |
| 24 | ENOPH1 | na | 23617 | -0.070 | -0.0432 | No |
| 25 | SRM | na | 24224 | -0.079 | -0.0395 | No |
| 26 | AHCYL2 | na | 24736 | -0.089 | -0.0306 | No |
| 27 | APIP | na | 25096 | -0.095 | -0.0158 | No |
| 28 | LDHA | na | 25221 | -0.097 | 0.0065 | No |
| 29 | DNMT3B | na | 25808 | -0.109 | 0.0186 | No |
| 30 | DNMT1 | na | 27273 | -0.142 | 0.0141 | No |
| 31 | SDS | na | 27287 | -0.142 | 0.0516 | No |
| 32 | LDHAL6B | na | 27633 | -0.151 | 0.0819 | No |
| 33 | CTH | na | 29164 | -0.196 | 0.0899 | No |
| 34 | CBS | na | 31015 | -0.265 | 0.1070 | No |
Table: GSEA details [plain text format]

  

Fig 2: KEGG\_CYSTEINE\_AND\_METHIONINE\_METABOLISM      
 Blue-Pink O' Gram in the Space of the Analyzed GeneSet

  

Fig 3: KEGG\_CYSTEINE\_AND\_METHIONINE\_METABOLISM: Random ES distribution      
 Gene set null distribution of ES for **KEGG\_CYSTEINE\_AND\_METHIONINE\_METABOLISM**
